# Entanglement structure of the two-component Bose-Hubbard model as a quantum simulator of a Heisenberg chain

**DOI:** 10.1038/s41598-019-45737-4

**Published:** 2019-07-01

**Authors:** I. Morera, Artur Polls, Bruno Juliá-Díaz

**Affiliations:** 1 Departament de Física Quàntica i Astrofísica, Facultat de Física, Universitat de Barcelona, E–08028 Barcelona, Spain; 2 Institut de Ciències del Cosmos, Universitat de Barcelona, ICCUB, Martí i Franquès 1, Barcelona, 08028 Spain; 3 ICFO-Institut de Ciencies Fotoniques, The Barcelona Institute of Science and Technology, 08860 Castelldefels, Barcelona, Spain

**Keywords:** Quantum simulation, Ultracold gases

## Abstract

We consider a quantum simulator of the Heisenberg chain with ferromagnetic interactions based on the two-component 1D Bose-Hubbard model at filling equal to two in the strong coupling regime. The entanglement properties of the ground state of the two-component Bose-Hubbard model are compared to those of the effective spin model as the interspecies interaction approaches the intraspecies one. A numerical study of the entanglement properties of the two-component Bose-Hubbard model is supplemented with analytical expressions derived from the effective spin Hamiltonian. When the pure ferromagnetic Heisenberg chain is considered, the entanglement properties of the effective Hamiltonian are not properly predicted by the quantum simulator.

## Introduction

The Bose-Hubbard model is almost ubiquitous nowadays in the interpretation of ultracold atomic gases experiments with optical lattices^1^. It provides the prime ingredient that allows ultracold atomic setups to mimic well-known many-body problems^1,2^. In particular, it makes these systems extremely competitive for building quantum simulators of a wide range of notably difficult physical problems^3,4^. A particularly relevant example is the use of a two-component Bose-Hubbard (TCBH) model as a quantum simulator of spin models^4–7^. As pointed out in refs^5,6^. different spins, e.g. 1/2, 1, etc, can be simulated depending on the filling factor of the two species in the chain. In the present article we concentrate on the specific configuration of filling one for both species, i.e. equal number of atoms of both species in the chain, in which case the TCBH can be mapped into a ferromagnetic Heisenberg spin −1 model^5^.

In general terms, a quantum simulator is defined as an experimentally feasible and versatile setup which is able to mimic a target Hamiltonian in different parameter regimes. In this way, for instance, the low energy physics of both the target Hamiltonian and the quantum simulator should be very similar. One could, however, be interested in other properties of the target system such as simulating the quantum correlations present in the ground state. The entanglement spectrum, characterizing the pairwise entanglement present in the system, provides a powerful witness of the presence of quantum correlations^8^. This seems a relevant goal for near-future applications of quantum entanglement to a variety of quantum technologies, see for instance^9^.

In this article we consider the question: To what extent does the quantum simulator exhibit similar entanglement properties to the simulated Hamiltonian? In particular, we focus on critical regimes where specific entanglement properties universally characterize the phase of the system. The analysis will be performed in the strongly interacting regime, where the interaction strength of both species is equal and much larger than the tunneling rate. We will study the entanglement properties of the system as the interspecies interaction is increased towards the point where all interactions are equal. In this way, the effective spin model goes from an anisotropic Heisenberg model into the isotropic Heisenberg one. Analytical results using perturbation theory will be complemented with numerical calculations using DMRG (density matrix renormalization group). In this way we can compare the entanglement present in the TCBH with that of the spin model, paying particular attention to the critical phases which appear in the latter.

## Model

We consider two bosonic species with contact-like interactions in a 1D optical lattice at zero temperature. We assume the system to be described by the TCBH Hamiltonian,

1 $$H=-\,t\sum _{i}\,\sum _{\alpha =A,B}\,({\hat{b}}_{i,\alpha }^{\dagger }{\hat{b}}_{i+1,\alpha }+{\rm{h}}.\,{\rm{c}}.)+\frac{U}{2}\sum _{i}\,\sum _{\alpha =A,B}\,({\hat{n}}_{i,\alpha }({\hat{n}}_{i,\alpha }-1))+{U}_{AB}\sum _{i}\,{\hat{n}}_{iA}{\hat{n}}_{iB},$$

where ${\hat{b}}_{i\alpha }$ (${\hat{b}}_{i\alpha }^{\dagger }$) are the annihilation (creation) bosonic operators at site *i* = 1, …, *L* for species *α* = *A*, *B*, and ${\hat{n}}_{i\alpha }$ are their corresponding number operators. We have assumed equal tunneling strength, *t* > 0, and equal repulsive intra-interaction strength, *U* > 0, for both components. For the rest of the work we set the energy scale to *t* = 1. The ground state (GS) of Eq. (1) in the strong-coupling regime (*U* ≫ *U*_*AB*_, *t*) is a Mott insulator (MI) with a total filling *ν* = *N*_*A*_/*L* + *N*_*B*_/*L* ≡ *ν*_*A*_ + *ν*_*B*_. In this work we fix *ν*_*A*_ = *ν*_*B*_ = 1.

We define the entanglement properties through the reduced density matrix obtained tracing out the right half of the system *ρ*_*L*/2_ = Tr_*R*_|*ψ*〉〈*ψ*|, where |*ψ*〉 is the ground state of the TCBH. The amount of entanglement is quantified with the von Neumann entropy *S*_*E*_ = −Tr*ρ*_*L*/2_log*ρ*_*L*/2_. Finally, the entanglement spectrum (ES)^10^ is defined in terms of *ξ*_*i*_ = −log*λ*_*i*_, where *λ*_*i*_ are the eigenvalues of the reduced density matrix.

## Results

### Perturbative regime

**Figure 1:**
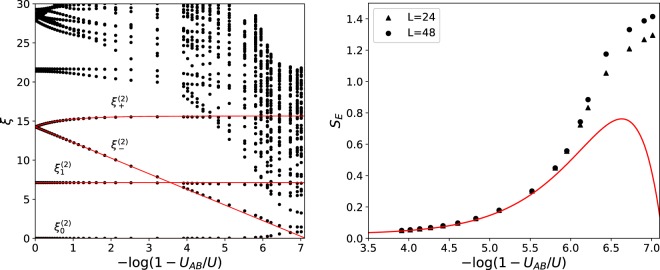
(Left panel) Entanglement spectrum of the TCBH (black dots) at fixed total size *L* = 48 as a function of *U*_*AB*_ for fixed value of *U* = 50 obtained with DMRG. Continuous red lines represent analytical results from Eqs (2) and (3), see text. The analytical model predicts a closing of the entanglement gap at *U*_*AB*_ = *U* − 2/*U*, which corresponds to −log(1 − *U*_*AB*_/*U*) ≈ 7.13. (Right panel) von Neumann entropy *S*_*E*_ as a function of *U*_*AB*_ for fixed *U* = 50 and for two different system sizes *L*. The solid red line is the analytical result obtained with perturbation theory.

In the strong-coupling regime (*U* ≫ *t*), the ES can be obtained perturbatively following^11^. In order to organize the ES we introduce the quantum numbers *δN*_*α*_ = *N*_*α*,*L*/2_ − *L*/2, with *α* = *A*, *B*. They measure the excess (*δN*_*α*_ > 0) or absence (*δN*_*α*_ < 0) of bosons with respect to the MI with filling *ν*_*A*_ = *ν*_*B*_ = 1 on the left subsystem (of size *L*/2). In Fig. 1 we report the obtained entanglement spectrum as a function of the interspecies interaction, *U*_*AB*_, for a fixed, large, value of *U* = 50. For *U*_*AB*_ = 0 the ES of the single-component Bose-Hubbard model is recovered, with clearly separated levels corresponding to different orders in perturbation theory on 1/*U*^11^. For non-zero values, *U*_*AB*_ > 0, some entanglement values exhibit an explicit dependence on this interaction. The entanglement values associated with *δN*_*A*_ = ±1; *δN*_*B*_ = 0 and *δN*_*A*_ = 0; *δN*_*B*_ = ±1 are given by,

2 $${\xi }_{1}^{(2)}=2\,\mathrm{log}\,U-\,\mathrm{log}\,2,$$

and do not show an explicit dependence on *U*_*AB*_ at the order studied. Furthermore, these ones are completely analogous to the first ones obtained for the single-component Bose-Hubbard. The lowest entanglement value associated with *δN*_*A*_ = *δN*_*B*_ = 0 gets a contribution ${\xi }_{0}^{(2)}=8/{U}^{2}$ due to the renormalization of the wavefunction.

Genuine second-order contributions are of two different kind: (+), corresponding to *δN*_*A*_ = *δN*_*B*_, which favor the movement of two different bosons through the boundary in the same direction and, (−), *δN*_*A*_ = −*δN*_*B*_, which favor the hopping of two different bosons through the boundary in opposite directions. Unlike ${\xi }_{1}^{(2)}$, these ones are absent in the single-component Bose-Hubbard model as they are directly related to the presence of two different components. Specifically, configurations with *δN*_*A*_ = −*δN*_*B*_ are associated to the phase separation of the two components through the boundary. An analytic formula can also be obtained,

3 $${\xi }_{\pm }^{(2)}=4\,\mathrm{log}(U)+2\,\mathrm{log}(1\pm {U}_{AB}/U)-\,\mathrm{log}\,4.$$

The two different branches, (+) and (−), have a very different behavior as *U*_*AB*_ is increased. The (−) one is seen to decrease as *U*_*AB*_/*U* → 1. This is expected as, in absence of tunneling, the system becomes highly degenerate at *U*_*AB*_ = *U*. We predict a closing of the entanglement gap – difference between the two lowest entanglement values^10^ – at *U*_*AB*_ = *U* − 2/*U*, see Fig. 1 (left panel). The fact that the entanglement gap closes is reflected in an increase of the von Neumann entropy *S*_*E*_, see right panel in Fig. 1. The analytical predictions are in very nice agreement with the DMRG calculations. Note also that the structure of the ES changes dramatically as we approach this point, with higher order processes becoming comparable to the lowest entanglement value. These higher order processes also show a logarithmic dependence as found for ${\xi }_{-}^{(2)}$ with a slope that indicates the order of the perturbation theory at which they are found. At this point we expect the system to enter in a critical regime. In this regime the von Neumann entropy *S*_*E*_ increases as we commented previously, but it also starts to depend on the system size, see Fig. 1.

### The TCBH as a quantum simulator

Interesting physics appears as (*U* − *U*_*AB*_) ~ 1/*U*. As discussed above, the system enters in a critical regime which cannot be described by the simple perturbation theory. Instead one has to consider a degenerate perturbation theory. For the general case of *n* atoms per site, the low-energy Hilbert space is described by an effective spin *S* ≡ *n*/2 where *A* and *B* are taken as a pseudo-spin 1/2. The effective Hamiltonian describing this low-energy spin subspace is given by superexchange processes at second-order in the hopping^5,7,12^

4 $${H}_{{\rm{eff}}}=-\,J\sum _{i}\,{{\bf{S}}}_{i}{{\bf{S}}}_{i+1}+D\sum _{i}{({S}_{i}^{z})}^{2},$$

where *J* = −4*t*^2^/*U* and *D* = *U* − *U*_*AB*_. Working with a fixed number of total bosons *ν*_*A*_ = *ν*_*B*_ = 1 maps in the spin picture to the sector with null total magnetization in the *z*-axis $\sum _{i}\,{S}_{i}^{z}=0$ and an on-site total spin *S* = 1.

The model (4) has been extensively studied^13–18^ and presents different phases depending on the ratio *D*/*J*. Here, we consider *D* ≥ 0. For *D*/*J* → ∞ (large −*D* phase) all spins tend to be in the zero *z*−projection and performing a perturbation theory calculation over this ground state at first-order in *J* leads to the same entanglement value ${\xi }_{-}^{(2)}$ previously found for the TCBH. At *D*/*J* ~ 1 the system enters in a critical *XY* ferromagnetic phase characterized by a conformal field theory (CFT) with central charge *c* = 1^15,17^. Finally, for *D* = 0 the system is in the isotropic point where its properties are governed by the *SU*(2) symmetry of the Hamiltonian (4).

In the limit of zero hopping, *t* = 0, and isotropic interactions, *U* = *U*_*AB*_, the spectrum of the Hamiltonian (1) has different degenerated subspaces separated by an energy scale of order *U*. The introduction of a small hopping *t*/*U* ≪ 1 and a small anisotropy |*U* − *U*_*AB*_| ≪ *U* breaks the degeneracy of these subspaces. Considering degenerated second-order perturbation theory in the hopping, one can write an effective Hamiltonian (4) which describes the degeneracy breaking inside the lowest energy subspace. In this way, the Hamiltonian (1) is mapped into the Hamiltonian (4). This is what allows one to term the TCBH a quantum simulator of the Heisenberg model. But what happens with observables? We deal with this question by using degenerated perturbation theory, see e.g.^19^. We split the TCBH Hamiltonian in two pieces, *H* = *H*_int_ + *H*_*t*_, corresponding to the interaction part, *H*_int_, and the tunnelling piece, *H*_*t*_, respectively. The lowest energy degenerated subspace of *H*_int_ is the one defining the subspace *P*, with energy ${E}_{0}^{p}$. The remaining states, which are separated by a gap proportional to *U*, define the *Q* subspace. In our case, the effective spin Hamiltonian (4) acts on the *P* space, while the TCBH acts on the complete Hilbert space, *P* ⊕ *Q*. Given the complete wave function |*ψ*〉, one can obtain the projection on the *P* subspace, $|{\psi }^{P}\rangle =\hat{P}|\psi \rangle$, where the projector reads, $\hat{P}=\sum _{\alpha \in P}\,|\alpha \rangle \langle \alpha |$, and |*α*〉 are eigenstates of *H*_int_. The inverse problem is formally written as $|\psi \rangle =\hat{{\rm{\Omega }}}|{\psi }^{P}\rangle$, where $\hat{{\rm{\Omega }}}$ is called wave operator. One can write expressions for $\hat{{\rm{\Omega }}}$ at a specific order in perturbation theory. Since the mapping between *H* and *H*_e*ff*_ has been obtained at second-order in the hopping, it is sufficient to express $\hat{{\rm{\Omega }}}$ at first-order in the hopping^19^,

5 $$\hat{{\rm{\Omega }}}=1+\sum _{\beta \notin P}\,\frac{|\beta \rangle \langle \beta |}{{E}_{0}^{P}-{E}_{0}^{\beta }}{H}_{t}$$

where ${E}_{0}^{\beta }$ are the eigenenergies of *H*_int_ corresponding to the eigenstate |*β*〉. In terms of the wave operator one can write the explicit expression ${H}_{{\rm{eff}}}=\hat{P}H\hat{{\rm{\Omega }}}\hat{P}$ which ensures that if *H*|*ϕ*〉 = *E*|*ϕ*〉 then *H*_eff_|*ϕ*^*P*^〉 = *E*|*ϕ*^*P*^〉, with $|{\varphi }^{P}\rangle =\hat{P}|\varphi \rangle$.

Finally, the reduced density matrix ${\hat{\rho }}_{L/2}$ associated to the groundstate of the complete Hamiltonian (1) |*ψ*_GS_〉 can be expressed in terms of the groundstate of the effective one (4) $|{\psi }_{{\rm{GS}}}^{P}\rangle$ as,

6 $${\hat{\rho }}_{L/2}={{\rm{Tr}}}_{R}\{|{\psi }_{{\rm{GS}}}\rangle \langle {\psi }_{{\rm{GS}}}|\}={{\rm{Tr}}}_{R}\{\hat{{\rm{\Omega }}}|{\psi }_{{\rm{GS}}}^{P}\rangle \langle {\psi }_{{\rm{GS}}}^{P}|{\hat{{\rm{\Omega }}}}^{\dagger }\}\ne {{\rm{Tr}}}_{R}\{|{\psi }_{{\rm{GS}}}^{P}\rangle \langle {\psi }_{{\rm{GS}}}^{P}|\}.$$

Thus, the question of how well the entanglement properties are reproduced in a quantum simulator is rewritten as: can $\hat{{\rm{\Omega }}}$ introduce some extra structure which affects the universal entanglement properties?

### Entanglement in the critical regime

**Figure 2:**
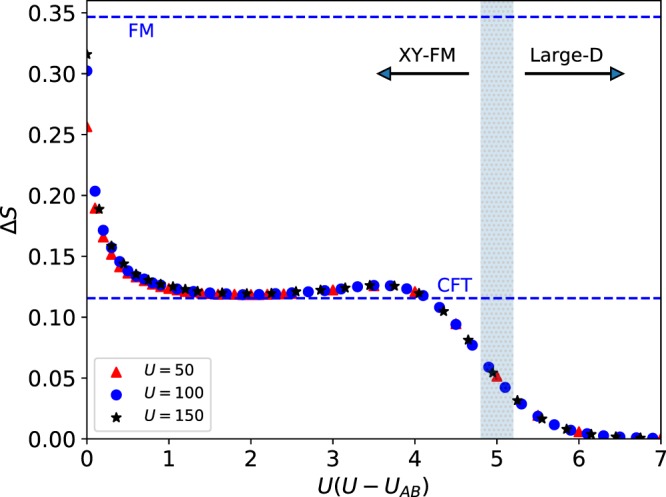
Main panel: Entanglement scaling Δ*S* of the TCBH as a function of the universal coupling *U*(*U* − *U*_*AB*_) for different values of the interaction *U* = 50,100,150 at fixed total system length *L* = 48. The upper dashed line represents the value predicted by the ferromagnetic (FM) Heisenberg model Δ*S* = (1/2)log2 and the lower one represents the CFT prediction Δ*S* = (*c*/6)log2 with central charge *c* = 1. The shaded area represents the region where the quantum phase transition between the large −*D* phase and the *XY*-FM phase is expected, extracted from^23^.

The scaling of the von Neumann entropy can be used to characterize the different phases of the system. From a CFT description this is a well-known result^20,21^ and the magnitude Δ*S* = *S*_*E*_(*L*) − *S*_*E*_(*L*/2) captures the scaling behavior properly^22^. Following the known behavior of the effective model, Eq. (4), one expects to go from Δ*S* → 0 in the large −*D* phase, to Δ*S* = (*c*/6)log2 in the critical *XY* phase with *c* = 1. This is exactly what is seen in Fig. 2, where we observe the crossover between the two regimes in the TCBH as we vary *U*(*U* − *U*_*AB*_) ~ *D*/*J*, with a very nice agreement with the results obtained for the effective spin model^23^. Furthermore, these results are mostly independent of *U*, for sufficiently large *U*. Therefore, we can conclude that the transition in the spin picture from a large −*D* to a critical *XY* ferromagnetic phase is captured by the transition observed in the TCBH. On the other hand, as *U* − *U*_*AB*_ → 0 a dependence on *U* starts to appear.

### Isotropic point

In the spin model, the isotropic point *D* = 0 is the end of the conformal line *c* = 1 describing the critical *XY* phase, and the system only exhibits scale invariance^24,25^. The *SU*(2) symmetry fully determines the ground state of the system, which is composed by a superposition of all the states belonging to the multiplet with maximum total spin. For a chain formed by *L* spins *S*, this multiplet is obtained by applying the lowering operator ${S}^{-}=\sum _{i}\,{S}_{i}^{-}$ to the fully polarized state $|{S}_{T}=SL,{S}_{T}^{z}=SL\rangle \equiv |F\rangle$. For specific sectors with fixed total magnetization ${S}_{T}^{z}\equiv SL-M$, the ground state of the system is $|{\psi }_{{\rm{GS}}}^{P}\rangle ={({S}^{-})}^{M}|F\rangle$, which is a superposition of all spin configurations in the chain satisfying that the total magnetization is ${S}_{T}^{z}$. Therefore, considering a bipartition of the system *A* of length *l* the ES is organized by eigenstates with well defined magnetization ${S}_{A}^{z}=Sl-m$ in the subsystem *A* with eigenvalues

7 $${\xi }_{m}(M,S,L,l)=-\,\mathrm{log}(\frac{(\begin{array}{l}2Sl\\ m\end{array})(\begin{array}{l}2S(L-l)\\ M-m\end{array})}{(\begin{array}{l}2SL\\ M\end{array})}),$$

with *m* = 0, …, 2*Sl*, which is a natural extension of the results presented in^24,26,27^.

From Eq. (7) an asymptotic expression for the von Neumann entropy *S*_*E*_ can be obtained considering *l* = *L*/2 and ${S}_{T}^{z}=0$

8 $${S}_{E}=\frac{1}{2}\,\mathrm{log}(\frac{SL\pi }{2})+\frac{1-\,\mathrm{log}\,2}{2}+{\mathscr{O}}({L}^{-1}).$$

Notice that in the thermodynamic limit any small anisotropy *D* > 0 will restore the conformal symmetry. For finite systems a smooth crossover between the CFT and the scale invariant prediction (8) is expected^28^, see Fig. 2. In this region is where a non-universal behavior of the TCBH model is observed and we obtain different scalings of *S*_*E*_ for different values of the interaction *U*. Furthermore, we observe that in the limit (*U* − *U*_*AB*_) → 0 the scaling mostly depends on the value of the interaction *U* and does not coincide with the value predicted by the spin model, Eq. (8).

**Figure 3:**
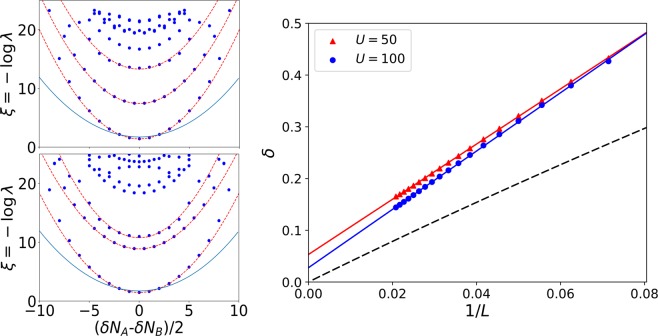
Left panel: Entanglement spectrum of the TCBH model (1) at *U* = 50 = *U*_*AB*_ (top) *U* = 100 = *U*_*AB*_ (bottom) and *L* = 48 as a function of the relative excess of bosons, blue circles, computed with DMRG. Red dashed lines represent parabolic fittings to the DMRG results and the continuous blue one is the analytic prediction given by the effective spin model. Right panel: Entanglement gap as a function of the inverse of the system length *L* for two different values of the interaction *U* considering the critical point *U*_*AB*_ = *U*. Continuous lines represent linear fittings and the dashed one is the analytic behavior predicted by the effective spin model.

In order to understand the dependence of the scaling of the entanglement entropy on the interaction *U* at *U* = *U*_*AB*_, we examine the ES of the TCBH model (1) and compare it with the analytical prediction for the spin model (7). The ES represented in Fig. 3 displays a parabolic dependence as a function of *δN*_*A*_ − *δN*_*B*_ (which is analogous to $\delta {S}^{z}={S}_{T}^{z}-{S}_{A}^{z}$ in the effective spin model). This parabolic dependence is also expected from the spin picture, see Eq. (7), but the curvature is considerably different. This curvature is directly related with the entanglement gap and one can observe that in both situations it depends linearly on the inverse of the system size *L*, see Fig. 3. But this linear dependence is different in the two models. Specifically, from the spin picture we obtain that *δ* → 4/*L*, so it closes in the thermodynamic limit *L* → ∞. Conversely, in the TCBH model the gap does not close in the thermodynamic limit for finite values of the interaction. Furthermore, the ES predicted by the spin model (7) has a well defined magnetization *δS*^*z*^, meaning that for each value of the magnetization there is a unique entanglement value ${\xi }_{\delta {S}_{z}}$. On the other hand, the ES of the TCBH model shows a richer structure with different parabolic envelopes for the same magnetization. Focusing on this extra structure we observe that the second parabolic envelope has associated a half-integer magnetization *δS*_*z*_, unlike the first one which has integer magnetization. This can be understood expanding the wave operator at first-order, see Eq. (5), $\hat{{\rm{\Omega }}}\simeq (1-{H}_{t}/U)$, where *H*_*t*_ is the hopping term of the Hamiltonian (1). The second envelope is obtained by the application of ${H}_{t}|{\psi }_{{\rm{GS}}}^{P}\rangle$ over the frontier which defines the bipartition of the system used to compute the ES. Therefore, these entanglement eigenstates correspond to having an extra particle or hole *δN* = ±1 for any of the two species which explains the half-integer nature of *δS*_*z*_. Notice that this component of the ground state wavefunction is reminiscent of the first entanglement eigenstates with eigenvalue ${\xi }_{1}^{(2)}$, Eq. (2). But now, due to the non-trivial entangled structure of the ground state, for each value of the subsystem magnetization we have this particle-hole excitation over the frontier which gives a large number of states, of order *L*. We have checked that the gap between the first two parabolic envelopes closes like 2log*U* and does not show an explicit dependence on the system length *L*.

**Figure 4:**
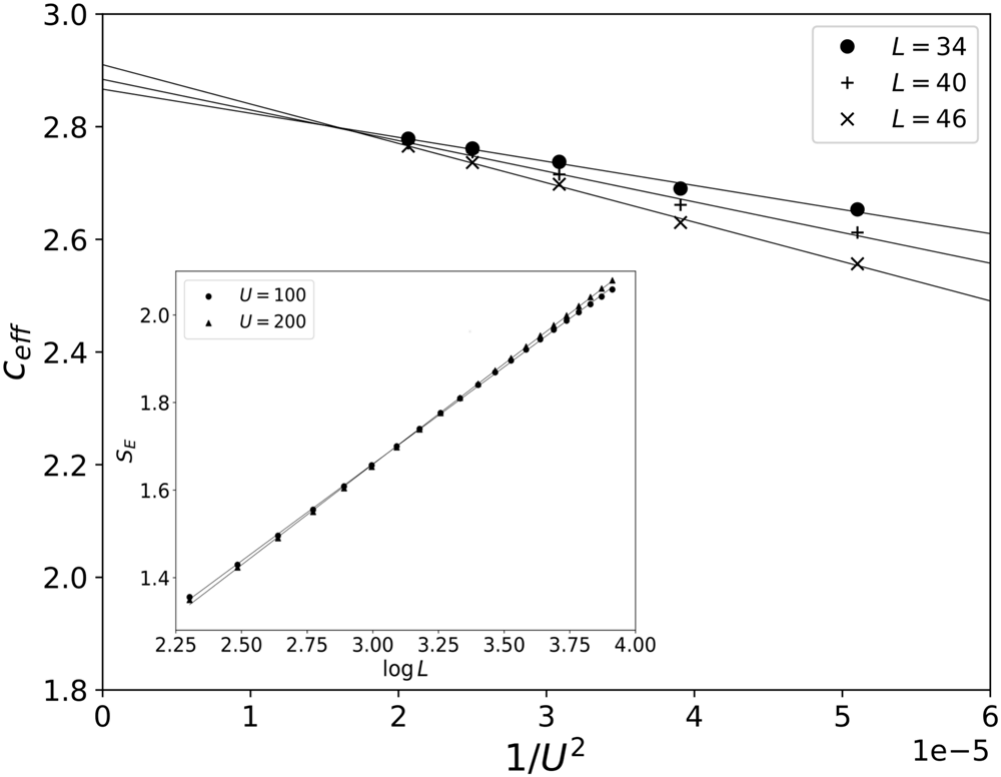
Main panel: Effective central charge (see main text) as a function of the inverse of the interaction *U* considering *U*_*AB*_ = *U* for different system sizes *L*. Inset: Entanglement entropy scaling for different values of the interaction. Continuous lines represent linear fittings.

The effect of including ${H}_{t}|{\psi }_{{\rm{GS}}}^{P}\rangle$ in the wavefunction has large effects on the von Neumann entropy. The main reason is that the number of entanglement states given by ${H}_{t}|{\psi }_{{\rm{GS}}}^{P}\rangle$ is of order *L* which is the same than the number of entanglement states in $|{\psi }_{{\rm{GS}}}^{P}\rangle$. Therefore, the contribution of both parts to the von Neumann entropy is log*L* and we can estimate the total contribution as *S*_*E*_ ∝ (1/2 − *A*/*U*^2^)log*L*, with *A* some constant value. In order to verify that, we define the slope *c*_eff_(*L*) = 6(*S*_*E*_(*L*) − *S*_*E*_(*L*_0_))/(log(*L*/*L*_0_)) with a reference size *L*_0_ = 50, for which finite size effects will be reduced^29^. In Fig. 4 we see that there is always a logarithmic behavior and the slope *c*_eff_(*L*) shows a clear dependence on 1/*U*^2^ which confirms our predictions.

## Conclusions

The extent to which a quantum simulator of a well-known spin system captures the entanglement properties of the ground state of the effective Hamiltonian has been scrutinized. We have considered the entanglement properties of the ground state of the two-component 1*D* Bose-Hubbard model in the strong-coupling regime for total filling *ν*_*A*_ = *ν*_*B*_ = 1. This model acts as a quantum simulator of the spin 1 Heisenberg model with ferromagnetic interactions. In the regime in which the spin system is in a critical *XY* phase (*U* − *U*_*AB*_ ~ *t*^2^/*U*) the two-component Bose-Hubbard model shows a universal (independent of the interaction *U*) scaling of the von Neumann entropy, which matches the CFT prediction expected for the effective spin system. On the other hand, we observe that this universality is lost as we approach the isotropic point *U* = *U*_*AB*_ where the effective spin model loses the conformal invariance. By comparing the ES of the quantum simulator with the effective spin model, which has been analytically obtained, we observe large discrepancies between the two of them for large values of the interaction *U*. In particular, magnitudes which should display a universal behavior (like the slope of the scaling in the entanglement entropy) strongly depend on the interaction *U*. This dependence has been analytically predicted constructing the wavefunction of the two-component Bose-Hubbard model using the wave operator.
